# Stromal *miR-20a* controls paracrine CXCL8 secretion in colitis and colon cancer

**DOI:** 10.18632/oncotarget.24495

**Published:** 2018-02-14

**Authors:** Steven A. Signs, Robert C. Fisher, Uyen Tran, Susmita Chakrabarti, Samaneh K. Sarvestani, Shao Xiang, David Liska, Veronique Roche, Wei Lai, Haley R. Gittleman, Oliver Wessely, Emina H. Huang

**Affiliations:** ^1^ Department of Stem Cell Biology and Regenerative Medicine, Lerner Research Institute, Cleveland Clinic, Cleveland, Ohio, USA; ^2^ Department of Cellular and Molecular Medicine, Lerner Research Institute, Cleveland Clinic, Cleveland, Ohio, USA; ^3^ Department of Molecular Cardiology, Lerner Research Institute, Cleveland Clinic, Cleveland, Ohio, USA; ^4^ Department of Colorectal Surgery, Cleveland Clinic, Cleveland, Ohio, USA; ^5^ Case Comprehensive Cancer Center, Case Western Reserve University School of Medicine, Cleveland, Ohio USA

**Keywords:** fibroblasts, miR-20a, CXCL8, colitis, colon cancer

## Abstract

Inflammatory bowel disease (IBD) affects one million people in the US. Ulcerative colitis (UC) is a subtype of IBD that can lead to colitis-associated cancer (CAC). In UC, the rate of CAC is 3-5-fold greater than the rate of sporadic colorectal cancer (CRC). The pathogenesis of UC and CAC are due to aberrant interactions between host immune system and microenvironment, but precise mechanisms are still unknown. In colitis and CAC, microenvironmental fibroblasts exhibit an activated, inflammatory phenotype that contributes to tumorigenesis accompanied by excessive secretion of the chemokine CXCL8. However, mechanisms regulating CXCL8 secretion are unclear. Since it is known that miRNAs regulate chemokines such as CXCL8, we queried a microRNA library for mimics affecting CXCL8 secretion. Among the identified microRNAs, *miR-20a/b* was further investigated as its stromal expression levels inversely correlated with the amounts of CXCL8 secreted and predicted fibroblast tumor-promoting activity. Indeed, *miR-20a* directly bound to the 3′UTR of *CXCL8* mRNA and regulated its expression by translational repression. *In vivo* co-inoculation studies with CRC stem cells demonstrated that fibroblasts characterized by high *miR-20a* expression had reduced tumor-promoting activities. These studies reveal that in stromal fibroblasts, *miR-20a* modulates CXCL8 function, therefore influencing tumor latency.

## INTRODUCTION

The pathogenesis of colitis-associated cancer (CAC) remains enigmatic. To date, both initiation of colitis, and its progression to dysplasia with a 3-5-fold increased rate of colorectal cancer [1], is likewise unclear. In addition, influences from genetics, the microbiome, and the immune system contribute both to the initiation of colitis and its progression towards CAC. Yet, the disease presentation and course are heterogeneous.

The inflammatory and cancer microenvironments both promote and prevent progression of disease. Within these microenvironments, fibroblasts had previously been believed to be passive members. However, in the last few decades, stromal fibroblasts have been discovered to be active members of these microenvironments [2], either preventing or promoting colitis and colorectal cancer. We have previously reported the contributions of inflammatory and cancer associated fibroblasts to colon cancer stem cell-initiated tumorigenicity [3]. These studies demonstrated that the secretome of stromal fibroblasts includes the inflammatory chemokine CXCL8. In this context, CXCL8 promotes angiogenesis and migration and may contribute to proliferation and tumorigenicity [4–6].

MicroRNAs (miRNAs) are short, non-coding RNAs regulating a myriad of cellular mechanisms including apoptosis, differentiation, and metabolism [7, 8]. In particular, they have been shown to contribute to many aspects of tumorigenesis. The miRNA expression profile is highly predictive for tumor progression [9] and targeting miRNAs has therapeutic benefits in chemoprevention or chemomodulation [10, 11]. The most canonical function of miRNAs is to regulate protein expression and mRNA stability by binding to complementary nucleotide sequences in the 3′UTRs. Most miRNAs are transcribed by RNA polymerase II as part of a much longer primary transcript (pri-miRNA), which becomes processed into the mature ~22 nt duplex miRNA by two consecutive RNA cleavages [12, 13]. The mature miRNAs are then loaded into the RNA-induced silencing complex (RISC), which binds to the 3′UTR of target mRNAs and induces translational inhibition or degradation. Target specificity is determined by the seed sequence (nucleotides 2 to 8 from the 5’ end of a miRNA) and is further strengthened by base pairing of flanking nucleotides [14, 15].

In this study we investigated whether miRNAs play a critical role in the tumor microenvironment by contributing to the transition of stromal fibroblast to colitis- and subsequent cancer-associated fibroblasts. In particular, we identified one miRNA, *miR-20a* that negatively regulates the expression of stromal fibroblast-derived CXCL8. We show that *miR-20a*, a member of the *miR-17* miRNA family, is part of a regulatory machinery that defines the pro-tumorigenic differentiation of stromal fibroblasts.

## RESULTS

### Differential secretion of CXCL8 from normal, colitic, and colon cancer-derived stromal fibroblasts is modulated by miRNAs

**Figure 1:**
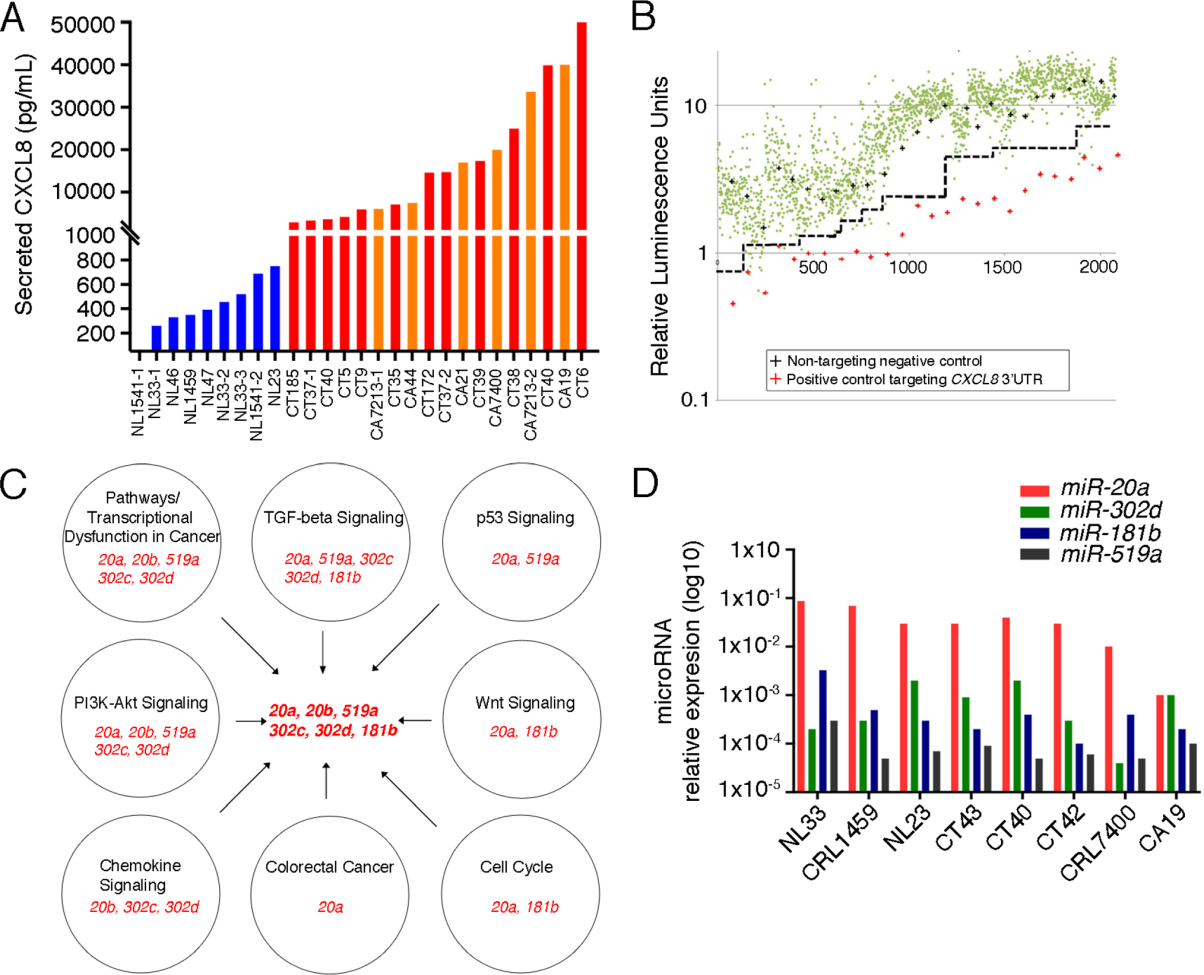
Identification of miRNAs Targeting *CXCL8* (**A**) ELISA analysis of CXCL8 levels present in the conditioned media of stromal fibroblasts isolated from normal (NL, blue, *N* = 8), colitic (CT, red; *N* = 9) and cancerous colons (CA, orange; *N* = 5). See Supplementary Table 2 for patient demographics. (**B**) Screening of a Miridian human mimic miRNA library using a *pMirGLO-CXCL8* 3′UTR luciferase construct in primary colitic fibroblasts. Green dots represent luciferase levels from the individual miRNA mimics, red pluses those of a siRNA targeting the 3′UTR of *CXCL8* and the black pluses those of a non-targeting negative control. Dashed black line delineates separation between mimics and the negative controls. (**C**) Pathway analysis of the identified miRNAs. Individual pathways and their associated miRNAs are depicted in the individual circles. miRNAs identified in these processes and selected for subsequent analysis are indicated in the center. (**D**) q-RT-PCR analysis of the miRNAs identified in (C) for their expression in multiple stromal fibroblasts. (NL: *N* = 3; CT: *N* = 3, CA: *N* = 2).

Previous reports indicate that enhanced secretion of CXCL8 occurs in the inflammatory and oncogenic colon microenvironments compared to grossly and histologically normal colon [3]. To further evaluate this we established primary cultures of normal colon, colitic colon, and cancerous colon associated fibroblasts and measured their CXCL8 concentrations in conditioned media (Figure 1A). As expected, the conditioned media demonstrate that colitis-associated (*N* = 11) and cancer-associated fibroblasts (*N* = 5) exhibit increased levels of CXCL8 compared to that of normal colon fibroblasts (*N* = 6).

Dysregulation of miRNAs have been implicated in the function of tumor-associated fibroblasts [16]. Thus, we queried whether posttranscriptional regulation by miRNAs is responsible for the modulation of CXCL8 levels. To test this hypothesis, we used the miRidian miRNA mimic library and screened over 2,500 miRNA mimics for repression of a luciferase reporter containing the 3′UTR of *CXCL8* mRNA. This approach identified 66 candidate miRNAs, of which 15 were verified in a secondary screen (Figure 1B and Supplementary Table 3). Next, we interrogated whether any of these miRNAs would have functions that align with their proposed tumor-promoting activity in colon cancer. Evaluating the candidate miRNAs using DIANA mirPath 2.0 [17] followed by KEGG pathway analysis [18] further reduced the number to 6 miRNAs belonging to four miRNA families (Figure 1C). Finally, we performed qRT-PCR using multiple colitis- and colon cancer-associated fibroblasts to determine whether their expression levels are sufficiently high to be relevant *in vivo* (Figure 1D). Based on these data we decided to focus on *miR-20a* and *miR-20b* as prime candidates to modulate CXCL8 expression during tumor progression. As both *miR-20a* and *miR-20b* bind to the same seed sequence and are thought to be identical in function, we focused on *miR-20a*.

### Regulation of *miR-20a* expression in stromal fibroblasts

**Figure 2:**
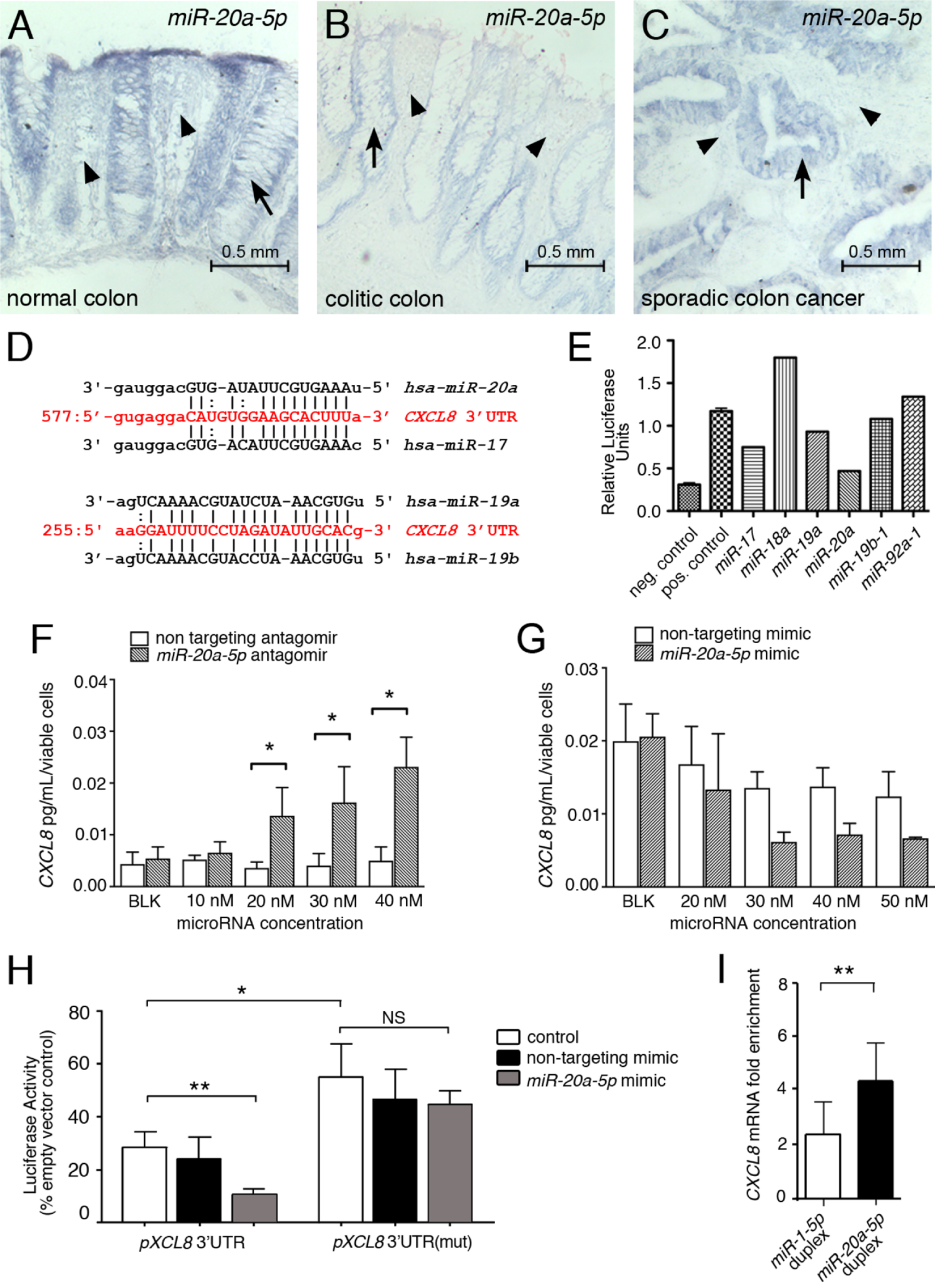
*miR-20a* directly modulates CXCL8 protein levels (**A**–**C**) *miR-20a* *in situ* hybridization on paraplast section of intestinal tissue from healthy individuals (A) and patients with colitis (B) and sporadic cancer (C). *miR-20a* (blue precipitate) is expressed in the epithelia (indicated by arrows), but also in the stroma between the intestinal crypts (indicated by arrowheads). The latter expression is markedly reduced in colitis and colon cancer. Scale bar 0.5 mm. (**D**) Sequence alignment of the *miR-20a/b* and *miR-19a/b-1* binding sites in the *CXCL8* 3′UTR. (**E**) Luciferase assay for the individual members of the *miR17~92* miRNA cluster regulating CXCL8. (**F**, **G**) Levels of secreted endogenous CXCL8 are modulated in a concentration dependent manner in *miR-20a* loss- and gain-of-function studies using antagomirs (F) and mimics (G). Asterisks indicate significance (2-way ANOVA with ^*^p < 0.05) (**H**) Luciferase assays using the wildtype *pMirGLO-CXCL8* 3′UTR luciferase construct and one with a mutated *miR-20a* binding site in the presence and absence of a non-targeting miRNA mimic or *miR-20a-5p*. Asterisks indicate significance (*t*-test with ^*^p < 0.05 and ^**^p < 0.01). (**I**) RNA immuno-precipitation using the miR-Trap system (Takara) in the presence of a *miR-20a* duplex and as a negative control a *miR-1* duplex, which does not have a detectable binding site in the *CXCL8* 3′UTR. Enrichment scores were calculated. Significance was assessed using *t*-test (^**^p < 0.005).

To confirm that the regulation of *miR-20a* is relevant *in vivo*, we queried human colon tissue sections by *in situ* hybridization for the expression of *miR-20a*. As shown in Figure 2A–2C, stromal *miR-20a* staining was stronger in normal colon, when compared to the stroma of colitic and cancerous colon. As previously reported [19], the epithelia in the colon cancer sections showed highly elevated *miR-20a* levels. Interestingly, this was not yet observed in the colitic epithelia, suggesting that the stromal changes in *miR-20a* expression precede the changes in the epithelia. Together, these data suggest that *miR-20a* levels are down regulated in the fibroblasts within the colitic field and is responsible for the up-regulation of CXCL8.

### *miR-20a* directly regulates CXCL8 translation

*In silico* analysis of the *CXCL8* mRNA identified a single *miR-20a* binding site in its 3′UTR (Figure 2D). *miR-20a* is encoded in the *miR-17~92* cluster, a critical regulator in multiple cancers [20–22]. The cluster contains six miRNAs belonging to three different miRNA families. Using the *CXCL8* 3′UTR reporter showed two *miR-17* family members, *miR-20a* and *miR-17*, as well as *miR-19a* - albeit to a lesser extent - repressed the luciferase activity (Figure 2E). The latter could be traced back to a distinct *miR-19* binding site in the 3′UTR of *CXCL8*, located approximately 300 base pairs downstream of the *miR-20* binding site (Figure 2D).

To further address the specificity of the *miR-20a*/CXCL8 interaction, we added an exogenous *miR-20a* mimic to *in vitro* cultures. Increasing the concentrations of the mimic decreased the levels of endogenously secreted CXCL8 accordingly, while a non-targeting mimic had no significant effects (Figure 2G). Conversely, blocking endogenous *miR-20a* using an antagomir resulted in a dose-dependent increase in CXCL8 secretion (Figure 2F). Mutating the *miR-20a* binding site in the *CXCL8* 3′UTR luciferase reporter rendered this construct unresponsive to the addition of exogenous *miR-20a* mimic (Figure 2H). Moreover, the mutated construct exhibited increased luciferase levels when compared to the un-mutated construct.

Finally, to determine, whether *miR-20a* targets the endogenous *CXCL8* mRNA, we performed RNA immuno-precipitation for GW182, a critical component of the RISC complex. As shown in Figure 2I, adding a synthetic *miR-20a* duplex significantly enriched immuno-precipitated *CXCL8* mRNA when compared to the addition of a *miR-1* duplex. Together, these data suggested that *miR-20a* is an important posttranscriptional regulator of *CXCL8* mRNA in colon interstitial fibroblasts.

### *miR-20a* expression levels are inversely correlated with the levels of secreted CXCL8

**Figure 3:**
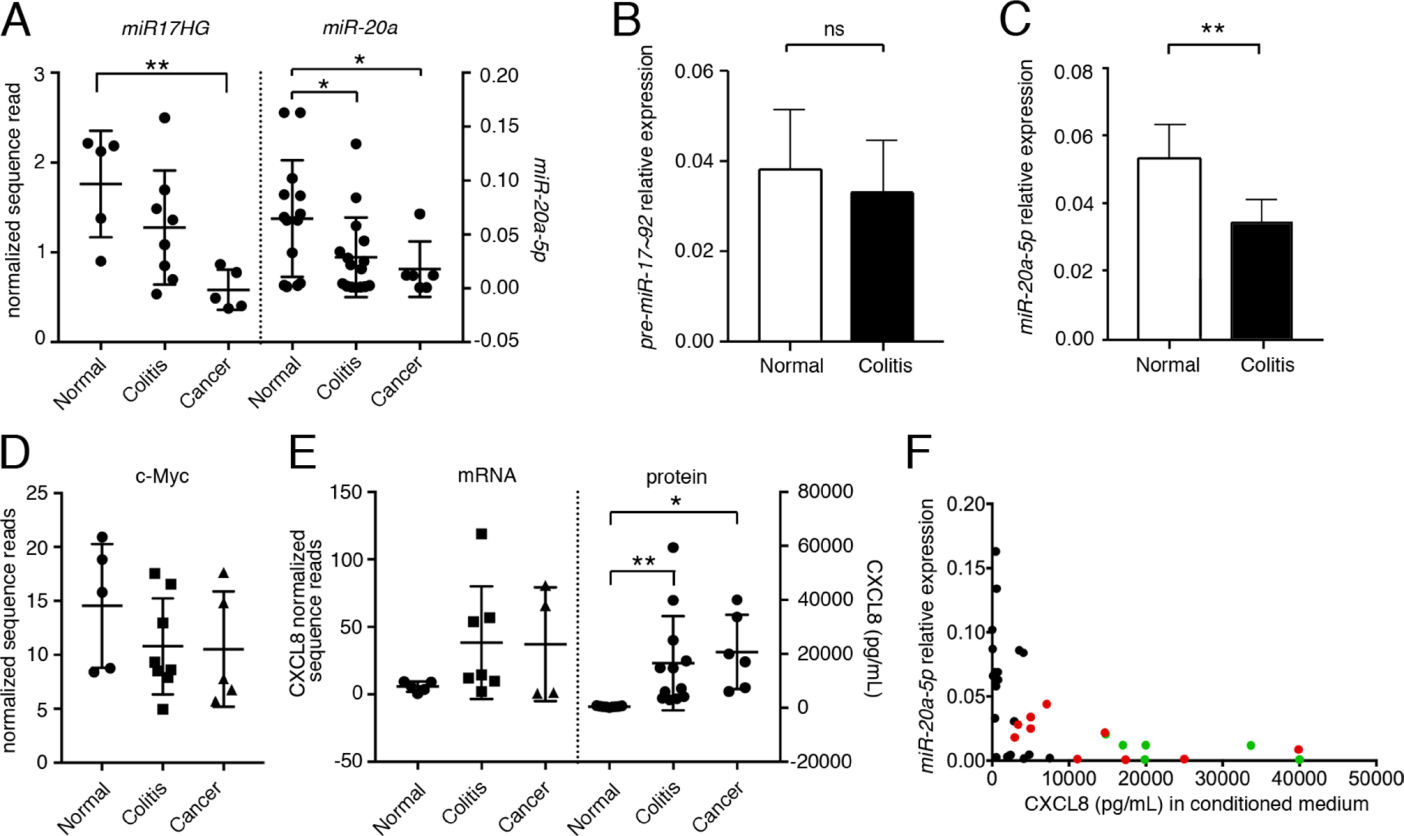
The expression of *miR-20a* is inversely related to secreted CXCL8 (**A**) Primary fibroblasts derived from normal, colitic and cancerous stroma were analyzed for the expression levels of the primary transcript encoding *miR-20a-5p* (*miR20HG*) and its mature form were quantified by mRNAseq and qRT-PCR, respectively. (**B**, **C**) qRT-PCR for the *pre-miR-20a-5p* (B) and *miR-20a-5p* (C) comparing normal and colitis-associated fibroblasts. Expression was normalized to *U18* RNA. (**D**) Expression of *c-MYC* by mRNAseq. (**E**) Levels of CXCL8 mRNA and protein by mRNAseq and ELISA. Statistical significance in (A–E) was determined using *t*-test; asterisks indicate significance (^*^p < 0.05 and ^**^p < 0.01). (**F**) Spearman’s correlation of *miR-20a* expression *vs.* secreted CXCL8 revealing a statistically significant inverse monotonic relationship (*p* < 0.001; r_s_ = –0.654). Normal colon fibroblasts: black, colitic colon fibroblasts: red, cancer-associated fibroblasts: green.

miRNA expression is regulated by the transcription of its primary transcript and the ensuing processing by the nuclear Drosha/DGCR8 and the cytoplasmic Dicer complex [12, 13]. qPCR analyses from normal, colitis-associated and cancer-associated fibroblasts demonstrated statistically significant differences in the levels of the mature *miR-20a* upon disease progression (Figure 3A). However, when we interrogated RNAseq data (GSE106119) for the expression of the mRNA encoding *miR-20a*, the *miR17HG*, a significant difference was only observed between normal and tumor-associated fibroblasts, while normal and colitis-associated fibroblasts merely exhibited a trend. This was confirmed by qRT-PCR analysis using primers detecting either the unprocessed *miR-20a* pre-miRNA or its mature counterpart (Figure 3B, 3C). The expression of the *miR17HG* is regulated by the transcription factor c-MYC [23]. Similar to the *miR17HG c-MYC* expression levels decreased upon severity of the disease (Figure 3D), suggesting that c-MYC may - at least partially - be responsible for the changes in *miR-20a* levels. Finally, we also compared *CXCL8* mRNA to protein levels (Figure 3E). As expected from a miRNA-mediated process, the CXCL8 protein levels were robustly altered, while the mRNA levels were reduced, but did not reach statistical significance.

Based on these results, we hypothesized that the levels of mature *miR-20a* and CXCL8 protein are reliable indicators for the pro-tumor promoting activity of the interstitial fibroblasts. To this end, we interrogated fibroblasts derived from normal, colitic and cancerous colon measured both for their cellular *miR-20a* levels and their amount of secreted CXCL8. As expected from our previous work, the levels of CXCL8 varied depending on disease state and statistically significant changes were seen between colitis and cancer compared to normal colon (Figure 3E). *miR-20a* levels showed the opposite trend being higher in normal cells than in colitis- or cancer-associated fibroblasts (Figure 3A). Most strikingly, measuring the monotonic relationship between both measurements using Spearman's correlation (Figure 3F) demonstrated a clear inverse relationship (r_s_ = –0.654, *p* < 0.001).

### Differential tumorigenesis of co-inoculations of *miR-20a*^high^ and *miR-20a*^low^ stromal fibroblasts with colon cancer stem cells

**Figure 4:**
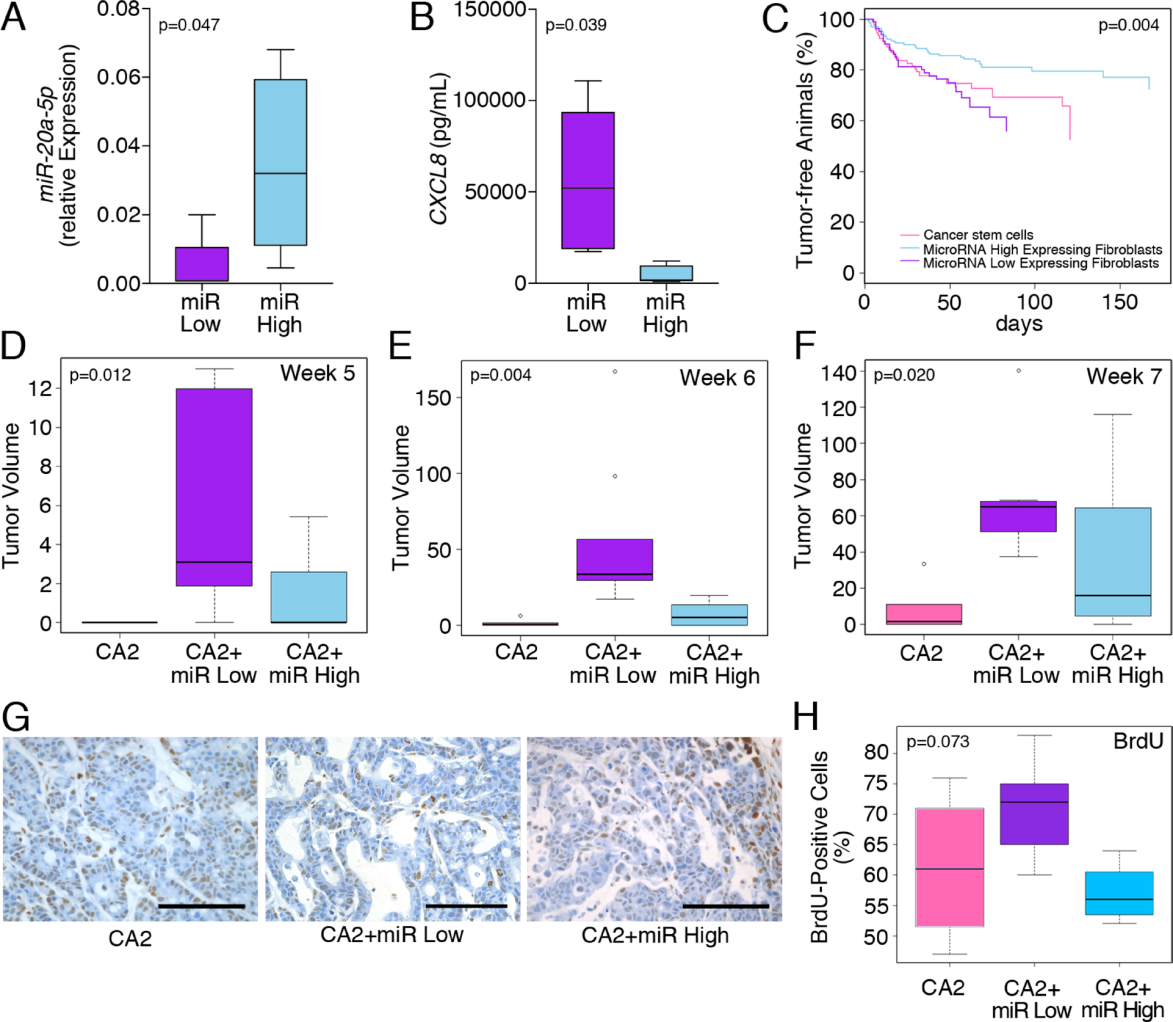
*miR-20a*/CXCL8 levels of co-inoculated stromal fibroblasts influence tumor latency (**A**, **B**) Confirmation of *miR-20a* and CXCL8 in the ‘miR High’ and ‘miR Low’ colitis-associated fibroblasts used for the *in vivo* tumorigenicity experiments. (**C**–**F**) Co-injection of colon cancer stem cells (CA2) with and without the miR High and miR Low colitis-associated fibroblasts significantly altered disease progression (C) and tumor volume (D–F) at 5 and 6 weeks, but not 7 weeks after inoculation. (**G**, **H**) Images and quantification of the tumors stained for BrdU incorporation upon resection. All xenografts were harvested, when the longest dimension was 5–7 mm. Statistics were preformed using ANOVA and significance levels are indicated in the individual panels. Each group contained *n* ≥ 9 animals.

The data so far demonstrated that changes in *miR-20a* levels are inversely related to the levels of the chemokine, CXCL8, which may influence the tumor promoting activity of colitis-associated fibroblasts. To test the *in vivo* significance of this observation, we chose to compare the effects of colitis-associated fibroblasts expressing high and low *miR-20a* levels (*miR-20a^high^* and *miR-20a^low^*) on colon cancer tumorigenicity. Expression of *miR-20a* as well as the concentration of secreted CXCL8 was confirmed by qRT-PCR and ELISA, respectively (Figure 4A, 4B). These fibroblasts were co-inoculated with colon cancer stem cells as a source of cancerous epithelia into the dorsal subcutaneous flanks of immunocompromised NSG mice. We used colon cancer stem cells since they are enriched in tumor initiating function [3]. The subcutaneous tumors were measured twice-weekly using calipers [3, 24–26]. Serial tumor measurements confirmed that tumor latency was decreased in mice injected with *miR-20a^low^* fibroblasts compared to those with the *miR-20a^high^* fibroblasts or colon cancer stem cells alone (Figure 4C). These differences in tumor volumes were most pronounced at weeks five and six post-inoculation (Figure 4D, 4E; *p* = 0.012 and *p* = 0.004, respectively). By week seven, the growth differences were still significant, however, the *miR-20a^high^* fibroblast/cancer stem cell co-injections revealed increased growth compared to the other groups (Figure 4F; *p* = 0.02).

When tumors had reached a length of 5–7 mm in at least one dimension, we injected mice with BrdU and harvested the tumors 3 hours later. This harvesting time point was chosen so that tumors were still actively proliferating, but did not yet develop central necrosis. In line with the tumor latency results, the BrdU staining showed a trend towards significance in the levels of BrdU incorporation with the *miR-20a^high^* fibroblast/cancer stem cell co-injections being the lowest (Figure 4G, 4H). In parallel, we performed immunohistochemistry for CXCL8 on the same tumor samples. However, this comparison was not significant (*p* = 0.523; Supplementary Figure 1A). Finally, since a downstream effector of the CXCL8 signaling pathway is the pro-angiogenic VEGF [6], we assayed the tumors for angiogenesis by MECA-32 immunostaining. However, like in the case of CXCL8, no differences could be detected between the three groups (*p* = 0.495; Supplementary Figure 1B). Together, these data support our observation that fibroblasts with different *miR-20a* levels alter tumor latency, but do not impact the tumor growth once it is established.

## DISCUSSION

Inflammation has long been identified as contributing to cancer, as the ‘wound that doesn't heal’ [27]. Here, we reveal that the tumor promoting activity of the inflammatory stroma is modulated, in part, by miRNAs (Figure 1). Specifically, our studies demonstrate that *miR-20a* directly regulates the amount of the proinflammatory chemokine CXCL8 secreted from the interstitial fibroblasts (Figure 2). Moreover, *miR-20a* and CXCL8 show an inverse relationship that models the transitions of the interstitial fibroblasts from normal to colitis-to tumor associated (Figure 3). We propose that low levels of *miR-20a* result in augmented tumorigenicity due to elevated levels of CXCL8. Conversely, tumor latency is increased, when *miR-20a* levels are elevated resulting in decreased CXCL8 protein secreted into the tumor microenvironment. Indeed, we can show that in *in vivo* experiments fibroblasts expressing either high or low levels of *miR-20a* impact tumorigenicity by influencing the tumor latency of co-inoculated sporadic colon cancer stem cells (Figure 4). The fact that once initiated, the tumors did not exhibit dramatic changes in proliferation or other tumor-associated parameters including angiogenesis suggest that the interstitial fibroblasts provide a supportive microenvironment for the initiation of the epithelial tumorigenicity, but do not alter the ultimate outcome.

miRNAs have been previously connected to colorectal cancer. For example, *miR-34b*/c and *miR-21* are strongly associated with poor prognosis [28]. Similarly, the *miR-17~92* cluster, which harbors the *miR-20a* studied here and is often referred to as ‘oncomir’ [29], has a pronounced role in cancer biology by promoting proliferation, inhibiting differentiation, increasing angiogenesis, and sustaining cell survival. In fact, the six miRNAs encoded in the *miR-17~92* cluster target many cancer relevant proteins such as VEGF and c-MYC [23, 30]. In colorectal oncogenesis the miR-17~92 cluster and in particular *miR-17*, which binds to the same seed sequence as *miR-20a*, is upregulated early on in tumor formation [31]. This upregulation is confined to the epithelia and has been closely linked to an auto-regulatory loop with c-MYC.

Interestingly, our data demonstrate that in the stromal fibroblasts the *miR-17~92* cluster and *miR-20a*, in particular play the opposite role, when compared to the epithelia. It is expressed highly in normal fibroblasts, but becomes down regulated in the course of their progression to a more tumor-promoting phenotype. This is due to changes in the transcription of the *miR-20a* host gene and may even involve c-MYC, a transcription factor that is highly associated with tumorigenesis [30]. As such, the fibroblasts and the epithelia appear to follow opposite paths. While the epithelia upregulate c-MYC and the *miR-17~92* cluster, the associated fibroblasts do exactly the opposite. How this is attained is currently unknown, but likely involves a yet-to-be-identified regulatory input that is specifically altered in colitis-associated fibroblasts. Its characterization will be very important to better understand how interstitial fibroblasts are undergoing pro-tumorigenic changes, of which *miR-20a* and CXCL8 are likely just a small aspect.

Based on the results presented here, miRNAs may play an integral part in this transformative process. miRNAs often exhibit rather modulatory functions, by changing e.g. the signal intensity, but not the signaling quality [32, 33]. In our study, CXCL8 expression is present in normal colon-associated fibroblasts, but is kept in check by the repressive activity of *miR-20a*. Only upon changes in the microenvironment *miR-20a* levels drop and the repression is relieved. These changes can be rather subtle initially, but worsen the pathogenicity over time. This process probably involves more than just *miR-20a/b*. It may include several of the other miRNAs that we identified as regulators of CXCL8 translation (Figure 1); it may also include other miRNAs that are up- or down-regulated in colitis-or cancer-associated fibroblasts, but do not impinge on *CXCL8* mRNA and were therefore not detected in our screen.

A slow differentiation process for the interstitial fibroblasts is in agreement with the long prodrome that usually exists for colitis-associated cancer. Risk of progressive disease from colitis to dysplasia to cancer occurs over decades and is dominantly associated with both duration and extent of disease. Thus, understanding the processes leading to or supporting carcinogenesis is therapeutically very important. It provides a rather wide window for less drastic treatments, which may delay/supplant the current intervention for medically refractory colitis and colorectal cancer, which is total proctocolectomy. Finally, understanding the pathogenesis of colitis-associated cancer and the contributions of the tumor microenvironment may be a paradigm to understand other inflammatory diseases such as Barrett's esophagus, for which the pathogenesis is likewise unknown.

In summary, our data support the emerging realization that therapeutic approaches targeting only epithelial cells are likely insufficient to prevent progression from colitis to dysplasia to CAC. Instead, a successful anti-CAC therapy also needs to involve strategies to prevent colitic fibroblasts from secreting tumor-promoting factors such as CXCL8 into the microenvironment.

## MATERIALS AND METHODS

### Human subjects

Collection and use of human tissues was approved by the Cleveland Clinic Institutional Review Board (IRB 13-1159). Tissues from human subjects were retrieved under pathologic supervision from the Cleveland Clinic. Normal colon tissues were obtained from areas at least 10 cm away from gross tumors. All human tissue samples were obtained from patient tissues that had undergone biopsy or surgery for resection of colon cancer or ulcerative colitis at the Cleveland Clinic. Informed consent in writing was obtained from each patient.

### Animals

All animal experiments were approved by the Cleveland Clinic IACUC Committee. Inbred NSG (non-obese diabetic IL-2) 4-6 week-old mice were used for these studies.

### Fibroblast isolation

Fibroblasts were isolated from primary tissues using previously described methodologies [3]. Briefly, after mincing, heated collagenase digestion on a shaking water bath was used and the filtrate was cultured on tissue culture plates in DMEM 10% FBS (Atlanta Biologicals) with 1% Pen/Strep (Gibco). Human colon fibroblasts cell lines CRL1541, CRL1459, and CRL7213 were obtained from the American Type Culture Collection (ATCC). Primary colon fibroblasts were isolated from human colon tissue was obtained with informed consent from patients undergoing surgery without preoperative treatment for sporadic colon cancer or colectomy for chronic ulcerative colitis. Primary fibroblasts were cultured from non-dysplastic sections of colon (normal), adenocarcinoma or colitic sections as previously described [3].

### Short tandem repeat analysis

Once established, primary isolates of fibroblasts and of the cancer stem cell epithelia were subjected to STR analysis (Duke University Genomics Laboratory) to establish their genetic identity. Prior to final use in assays, a second analysis was completed to confirm the genetic identity of the isolate, and compared it to the original STR for uniqueness (Supplementary Table 1).

### CXCL8 ELISA

To quantify the concentration of CXCL8 from the fibroblast conditioned media, 10^5^ stromal fibroblasts were cultured in 1 ml of DMEM base media for 24 h in the absence of serum. The conditioned media was harvested and subjected to quantification per the CXCL8 ELISA manufacturer's instructions (Raybiotech).

### Reporter constructs

The entire 3′-UTR of human *CXCL8* mRNA (1,350 bps) was cloned into the *pMIRGLO* vector (Promega) using *Sac*I and *Xba*I restriction enzymes. This cloning strategy placed the *CXCL8* 3′-UTR immediately downstream of the firefly luciferase gene driven by the PGK promoter. Constitutively expressed *Renilla* luciferase served as a normalizing control. To confirm the *in silico* predicted *miR-20a* recognition sequence within the *CXCL8* 3′-UTR, the nucleotides corresponding to the seed sequence (5′-AAG CAC TTT A-3′; bases 591-600) were mutated by site-directed mutagenesis to 5′-TTC GAC TTT A-3′. The *miR-20a* seed sequence prediction and its mitigation was performed using the PITA microRNA target prediction algorithm [34].

### Screening of human mimic microRNA library

A human mimic microRNA library consisting of 2080 unique siRNA corresponding to known human microRNAs (MiRIDIAN, ThermoFisher) was used for screening microRNA that interact with the 3′-UTR of human *CXCL8* mRNA. Immortalized colon fibroblasts (CT5 SV40) were seeded at 8000 cells per well in 96-well plates the day before transfection. These cells were immortalized via lentiviral transduction of hTERT SV40 (kind gift from Rosa Hwang, MD, MD Anderson). The next day each well of cells was co-transfected with unique library microRNA mimics (50 nM/well) and *pMIRGLO-CXCL8-3′UTR* plasmid using Dharmafect Duo (GE Dharmacon) according to the manufacturer protocol. After 48 hours luminescence was measured using DualGlo Luciferase Assay System (Promega) with a Spectra II plate reader (Molecular Devices). A non-targeting negative control and a positive control in form of a siRNA targeting the 3′UTR of human *CXCL8* mRNA (5’-AUU CUA GCA AAC CCA UUC AUU-3′, Dharmacon) were included for each individual mimic 96-well plate screen.

### microRNA target prediction by *in silico* analysis

miRNA pathway analysis and the identification of miRNA binding sites in the *CXCL8* 3′ UTR was performed using four prediction algorithms, DIANA Tools (http://diana.imis.athena-innovation.gr/DianaTools/index.php), microRNA.org, mirWALK (http://zmf.umm.uni-heidelberg.de/apps/zmf/mirwalk2/) and Target Scan (http://www.targetscan.org/).

### Luciferase knockdown assay

Human colon fibroblasts were seeded in a 96-well plate at a concentration of 1 × 10^4^ cells per well. Twenty-four hours after seeding, fibroblasts were transfected with either parental *pMIRGLO* plasmid, wild type *pMIRGLO-CXCL8-3′UTR* or mutagenized *pMIRGLO-CXCL8-3′UTR(mut)* plasmid using Lipofectamine 2000. After 48 hours, luciferase activity was measured with DualGlo (Promega) and normalized to Renilla activity using a Spectra II plate reader (Molecular Devices).

### RNA isolation, RNAseq and real-time qPCR

Total RNA was extracted using the miRNeasy kit method (Qiagen). Purity and integrity of the extracted RNA was assessed using the Aligent Bioanalyzer, and only samples with RIN values greater than 8.0 were used for gene expression analysis. Total RNA was processed by Beckman-Coulter Genomics for next generation sequencing using the TruSeq Total RNA kit (Illumina). Total RNA of each sample was depleted of ribosomal RNA using biotinylated oligos, combined with Ribo-Zero rRNA removal beads and subsequent fragmentation of RNA using divalent cations under elevated temperature. After reverse transcription the double stranded cDNA and ligation of sequencing adapter, the cDNA products were purified and enriched with PCR to create the library. Sequencing reads (single 100-bp) generated from the Illumina HiSeq 2500 platform were assessed for quality using FastQC (Tuxedo package retrieved from http://cufflinks.cdcd.umd.edu/igenomes.html). The reads were trimmed for adapter sequences using Trim Galore (https://github.com/FelixKrueger/TrimGalore). Alignment was to the human genome (GRCh19 assembly) using the Tuxedo package [35] followed by analysis for differential expression using a significance cut-off of a false discovery rate (FDR) <0.05 and a log2 fold change of greater than 1.5 or less than –1.5. All files are available at GEO (Accession Number: GSE106119).

The TaqMan microRNA qPCRs were used to perform reverse transcription and quantification of microRNAs. Primer sets specific for the mature and the unprocessed form of *hsa-miR-20a-5p* as well as small RNA *U18* as endogenous control were obtained from Fisher Scientific. miRNA expression was normalized to *U18*.

### *In situ* hybridization

*In situ* hybridization on paraplast sections was performed as previously described [36] using a digoxygenin-labeled *miR-20a* LNA-modified probe from Exiqon.

### miRNA mimic and antagomir experiments

Normal adult human colon fibroblasts were seeded at a density of 40,000 cells/well in a 24-well plate in DMEM with 2% fetal bovine serum without antibiotics. The following day cells (approximately 80% confluent) were transfected with either the *miR-20a* or a control miRNA mimic at concentration ranges between 0-50 nM using Lipofectamine RNAiMAX reagent according to the manufacturer's instructions (ThermoFisher Scientific). On day 3 after transfection, the medium was changed to plain basal DMEM, which was collected 24 hours later to measure the amount of secreted CXCL8 by ELISA. The adherent cells were washed 2 times with pre-warmed PBS and the number of viable cells in each well was estimated using the CellTiter-Glo cell viability assay (Promega). The antagomir experiments were performed in identical fashion using a *miR-20* antagomir or a non-targeting control (Exiqon).

### RNA immunoprecipitation

Verification of as *CXCL8* mRNA as the target of endogenous *miR-20a* was performed by RNA immunoprecipitation using the MirTrap System following the manufacturer's instructions (Takara). Human colon fibroblasts plated in 10 cm dishes (90% confluency) were co-transfected with 500 pmoles of the *miR-20a-5p* duplex or the *miR-1-5p* duplex and 30 μg of the pMirTrap plasmid using Lipofectamine 2000. The RNA duplexes were synthesized by IDT (*miR-20-5p*: passenger strand 5′phos-CUA CCU GCA CUA UAA GCA CUU CAA G-3′ and guide strand 5′phos-UAA AGU GCU UAU AGU GCA GGU AGC G-3′, *miR-1-5p*: passenger strand 5’phos-AUG GGC AUA UAA AGA AGU AUA UAG-3′ and guide strand 5′phos-ACA UAC UUC UUU AUA UGC CCA UCG-3′). *CXCL8* and *GAPDH* mRNA levels were measured by qRT-PCR using SYBR Green and enrichment scores were calculated as describe in the user manual.

### Co-injection tumorigenesis experiments

Xenografts consisting of cancer stem cell line known as CA2 enriched using the Aldefluor^®^ assay [3, 25]. One hundred cancer stem cells (CA2) were inoculated in the absence (baseline) or presence of 1000 stromal fibroblast cells in a 50:50 mixture of Matrigel^®^ (BD) into the subcutaneous right dorsal flank of NSG mice as previously described [3, 24–26]. The cells were re-suspended in media at concentrations of 2 × 10^3^ cells/ml and 2 × 10^4^ cells/ml, epithelia and stromal fibroblasts, respectively. At the time of stromal cell harvest, conditioned media and cells were retained to confirm the *miR-20a* miRNA levels and CXCL8 concentration. Tumors were measured twice per week using calipers for palpable tumors. Tumor volumes were calculated using the formula: volume = width^2^ × length (mm, length as the longer measurement). Tumors were harvested when additive total dimensions were approximately 15 mm, both to avoid central necrosis of the tumor and to minimize undue distress to the murine host.

Tumor growth curves were plotted using Kaplan-Meier survival curves, and differences in growth curves was assessed using the log-rank test. Tumor growth was assessed at week 5, week 6, and week 7, and differences between treatment groups at each time point was conducted using ANOVA. Significance was determined at the 0.05 level. ANOVA was used to assess the mean difference in positive percentage between the BrdU, CXCL8 and MECA-32 measurements in three treatment groups: cancer stem cells (CA2), cancer stem cells co-inoculated with *miRNA-20a* low expressing fibroblasts (CA2+miR Low), and cancer stem cells co-inoculated with *miRNA-20a* high expressing fibroblasts (CA2+miR High).

### Immunohistochemistry

Mice received IP injections of BrdU (10 μg/kg) 3 hours prior to harvest. Tumor sections were fixed in 4% paraformaldehyde. Tumor sections were deparaffinized in xylene and rehydrated in descending percentages of ethanol. Tissues were fixed in 4% PFA for 24 hours; 4 μm sections were cut and stained for BrdU incorporation using a commercially available kit (BrdU Staining Kit, Invitrogen) following the manufacturer's instructions. Five random images were documented under bright field using a Leica DMIRB microscope at 40× magnification. Both BrdU positive nuclei and total nuclei for each image were quantified using ImageJ software.

For CXCL8 staining slides were incubated with the anti-CXCL8 antibody (MAB208, R&D Systems) and visualized using the OmniMap anti-Mouse HRP secondary (760-4310, Ventana) and the ChromoMap DAB detection kit (760-159, Ventana). Slides were counterstained with hematoxylin and bluing. Representative images were documented at 40× using a Leica upright microscope. Sections from at least 9 animals per treatment group were evaluated, counting over 3,000 epithelial cells per group. Positivity was quantified as the ratio of DAB-stained colonic epithelia to the total number of nuclei-positive colonic epithelia.

To determine tumor vessel density, deparaffinized, 4 mm paraffin sections from tumor xenografts underwent a 20 minute, 95° C heat retrieval step using Target Retrieval Solution (DakoCytomation). Following water and buffer washes, the sections were blocked for 60 minutes in 2% horse serum followed by a 24 hour incubation at 4° C with a rat anti-MECA 32 (mouse pan-endothelial) monoclonal antibody (1:10; BD Pharmingen) and visualized using an anti-Rat Alexa Fluor 594-coupled secondary antibody (1:500; Invitrogen). Slides were mounted with VectaShield (Vector Laboratories) containing DAPI and five random images per section were documented at 40× magnification. Vessel density was quantified using grid overlay method (ImageJ; grid plugin; 70 μm^2^ squares) and normalized to mm^2^.

## SUPPLEMENTARY MATERIALS FIGURE AND TABLES





## Abbreviations

CAC: colitis-associated cancer
IBD: inflammatory bowel disease
UC: ulcerative colitis
