# Supplementary material for: Stromal miR-20a controls paracrine CXCL8 secretion in colitis and colon cancer
Source: Oncotarget. 2018 Feb 14;9(16):13048–59. doi: 10.18632/oncotarget.24495 (PMC5849194; doi:10.18632/oncotarget.24495)
Supplement: Supplementary file 1 [file oncotarget-09-13048-s001.pdf]

Stromal *miR-20a* controls paracrine CXCL8 secretion in colitis and colon cancer

SUPPLEMENTARY MATERIALS

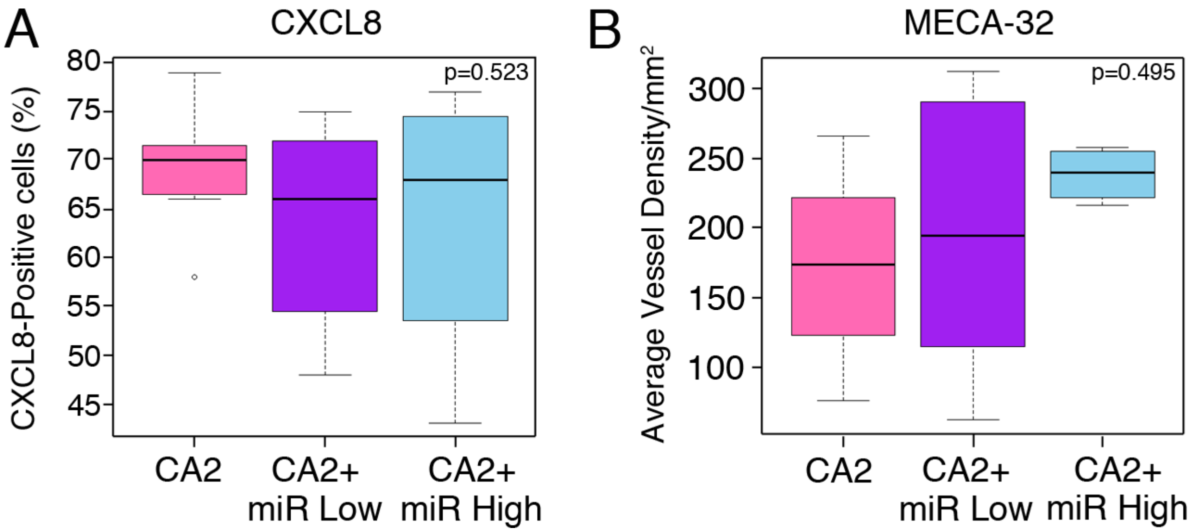

Supplementary Figure 1: Quantification of CXCL8 and MECA-32 immunohistochemistry of xenograft tumors. Quantification of CXCL8 (**A**) and MECA-32 (**B**) immunostaining of harvested co-inoculation xenografts consisting of cancer stem cells (CA2) with either *miR-20a*^{high} or *miR-20a*^{low} colitis-derived fibroblasts. Statistics were preformed using ANOVA and significance levels are indicated in the individual panels. Each group contained $n \geq 9$ animals.

Supplementary Table 1: Patient demographics

Patient ID	Disease	Pathology (overall)	M/F	Race	Disease Duration (Years)	Age at resection
NL 23	Colon cancer	Normal Margin	F	White	NA	Unknown
NL 33	Colon cancer	Normal Margin	M	White	NA	Unknown
NL 46	Colon cancer	Normal Margin	F	White	NA	63
NL 47	Colon cancer	Normal Margin	M	White	NA	72
CT 4	Colitis	Colitis and Rectal Cancer	M	White	13	36
CT 5	Colitis	Colitis	M	White	2	20
CT 6	Colitis	Colitis	F	White	1	59
CT 9	Colitis	Pancolitis	F	White	1	30
CT 35	Colitis	Pancolitis	F	White	30	47
CT 37	Colitis	Chronic active colitis	M	White	Unknown	36
CT 38	Colitis	Indeterminate colitis	F	White	2	64
CT 39	Colitis	Diffuse Colitis	M	White	7	57
CT 40	Colitis	Diffuse Pancolitis	M	White	2	32
CT 42	Colitis	Diffuse chronic active colitis	F	White	3	49
CT 43	Colitis with high-grade dysplasia	Diffuse chronic active colitis with low-grade dysplasia	M	Asian	10	53
CA 6	Colon Cancer	Stage 2	M	White	NA	Unknown
CA 19	Colon Cancer	Stage 1	F	White	NA	62
CA 21	Rectal Cancer	Stage 2	M	White	NA	Unknown
CA 22	Colon Cancer	Stage 3	M	White	NA	Unknown
CA 44	Colon cancer	Stage 1	F	White	NA	30

Supplementary Table 2: Short-tandem Repeat fingerprinting

Sample Name	D13S317	D16S539	CSF1PO	TH01	vWA	D21S11	D7S820	D5S818	TPOX	Amelogenin
CT6 Fb	11, 12	11, 12	12	6, 8	14, 16	28, 30	8, 11	11, 12	8, 9	X, Y
CT42	11, 13	11, 13	11, 13	7, 9.3	18	31, 33.2	10	12	8, 9	X, Y
CRL1459	12	12	8	6, 7	15, 17	29, 30	8	12	8, 11	X, Y
CRL7213	11, 12	11, 12	10, 12	8, 9	17, 18	28, 32.2	8, 14	11	8, 9	X
CAF58	12	12	10, 12	6, 9.3	16, 17	29, 32.2	8, 9	11, 12	8	X
33NL	11	11	11, 12	8, 9.3	15, 17	29, 32.2	10, 12	9, 11	8	X, Y

Unique identities of isolates used in studies were confirmed for the date presented in Figures 3 and 4.

Supplementary Table 3: Identification of miRNAs regulating the CXCL8 3′UTR. See Supplementary_Table_3
