# Supplementary material for: Stromal miR-20a controls paracrine CXCL8 secretion in colitis and colon cancer
Source: Oncotarget. 2018 Feb 14;9(16):13048–59. doi: 10.18632/oncotarget.24495 (PMC5849194; doi:10.18632/oncotarget.24495)
Supplement: Supplementary file 2 [file oncotarget-09-13048-s002.docx]

**Supplementary Table 3: Identification of miRNAs Regulating the *CXCL8* 3’UTR.**

| **Positive miRNAs** | | |
| --- | --- | --- |
| **Primary Screen** | | **Secondary Screen** |
| **Plate 1** | mir-20a-5p | mir-20a-5p |
|  | mir-92a-3p | mir-20b-5p |
|  | miR-32-5p | mir-302c-3p |
|  | mir-148a-3p | mir-372 |
| **Plate 2** | mir-223-3p | mir-573 |
|  | mir-302b-5p | mir-1273e |
|  | let-7i-5p | mir-3616-5p |
|  | hsa-miR-302c-3p | mir-4312 |
| **Plate 3** | mir-302d-3p | mir-4642 |
|  | mir-372 | mir-519a-3p |
|  | miR-20b-5p | mir-302d-3p |
| **Plate 4** | mir-503-5p | mir-3620-5p |
|  | mir-518b | mir-3689a-3p |
|  | mir-518d-3p | mir-181b-3p |
|  | mir-519a-3p | mir-4452 |
| **Plate 5** | mir-618 |  |
|  | mir-620 |  |
|  | mir-92b-3p |  |
|  | mir-573 |  |
|  | mir-622 |  |
| **Plate 6** | No positives |  |
| **Plate 7** | mir-92b-5p |  |
|  | mir-7-2-3p |  |
| **Plate 8** | mir-33b-3p |  |
| **Plate 9** | mir-93-3p |  |
| **Plate 10** | mir-513c-5p |  |
|  | mir-320c |  |
|  | mir-1264 |  |
| **Plate 11** | No positives |  |
| **Plate 12** | mir-1273e |  |
|  | mir-3616-5p |  |
|  | mir-3689a-3p |  |
| **Plate 13** | mir-3692-3p |  |
|  | mir-3117-3p |  |
|  | mir-3941 |  |
|  | mir-3935 |  |
|  | mir-3942 |  |
| **Plate 14** | mir-3133 |  |
|  | mir-466 |  |
| **Plate 15** | mir-4312 |  |
| **Plate 16** | mir-181b-3p |  |
| **Plate 17** | mir-548ao-5p |  |
|  | mir-5008-5p |  |
|  | mir-3619-3p |  |
|  | mir-5091 |  |
|  | mir-5094 |  |
|  | mir-4418 |  |
| **Plate 18** | mir-4452 |  |
| **Plate 19** | mir-4642 |  |
| **Plate 20** | mir-4655-5p |  |
|  | mir-4662a-5p |  |
|  | mir-4656 |  |
|  | mir-4663 |  |
|  | mir-4659a-3p |  |
|  | mir-4661-5p |  |
| **Plate 21** | mir-203b-3p |  |
|  | mir-4475-3p |  |
|  | mir-4756-3p |  |
| **Plate 22** | mir-4764-3p |  |
|  | mir-4759 |  |
|  | mir-4764-5p |  |
|  | mir-4802-5p |  |
|  | mir-4798-3p |  |
| **Plate 23** | No positives |  |
| **Plate 24** | mir-548ax |  |
|  | mir-5586-5p |  |
|  | mir-5697 |  |
| **Plate 25** | mir-3620-5p |  |
| **Plate 26** | No positives |  |

The miRIDIAN microRNA Library (Bundle 19.0, ThermoFisher) was screened using the pMIRGLO-CXCL8-3’UTR. Those microRNAs revealing luminescence above the control threshold are indicated
